# Successful treatment of obscure gastrointestinal bleeding with intraoperative enteroscopy

**DOI:** 10.1055/a-2107-2889

**Published:** 2023-06-27

**Authors:** Mohamed G. Shiha, Foong Way David Tai, Hey-Long Ching, Arun Loganathan, David S. Sanders

**Affiliations:** 1 Academic Unit of Gastroenterology, Sheffield Teaching Hospitals and The University of Sheffield, Sheffield, United Kingdom; 2 Department of General Surgery, Sheffield Teaching Hospitals, Sheffield, United Kingdom

A 51-year-old man was referred to our institution with persistent iron deficiency anemia. Initial gastroduodenoscopy and colonoscopy at his local hospital were unremarkable. A subsequent small bowel capsule endoscopy revealed a distal small bowel polyp with evidence of fresh bleeding ( Fig. 1 ). A triple-phase abdominal computed tomography scan confirmed a 13-mm vascular polypoid lesion within the distal small bowel, with hyperenhancement on the arterial phase ( Fig. 2 ). After a discussion in a multidisciplinary team meeting, it was decided that surgical resection guided by intraoperative enteroscopy to localize the lesion would be the most appropriate course of action.

**Fig. 1:**
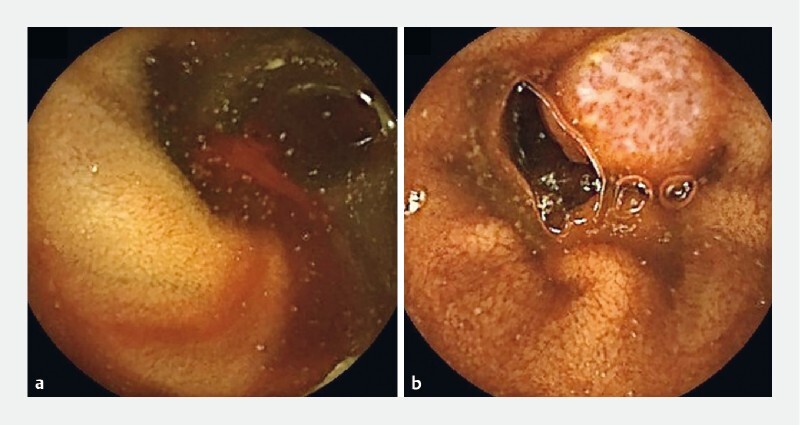
Small bowel capsule endoscopy images. **a** Fresh bleeding. **b** Polypoid lesion in the distal small bowel.

**Fig. 2:**
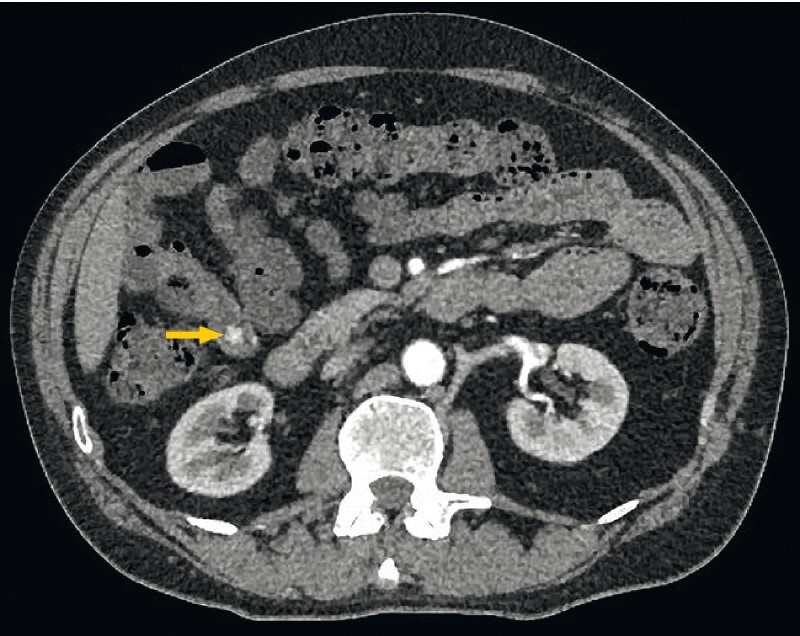
Abdominal computed tomography scan showing a polypoid lesion within the distal small bowel with hyperenhancement on the arterial phase.

During intraoperative enteroscopy, an actively bleeding polyp was detected in the distal ileum ( Video 1 ), and a limited small bowel resection was performed without complications ( Fig. 3 ). The patient was discharged 2 days after the surgery. Histological examination of the resected specimen showed a nodular area of ulceration lined with prominent granulation tissue. However, the cause for this ulceration was not histologically identified.

**Video 1** The intraoperative enteroscopy procedure.


**Fig. 3:**
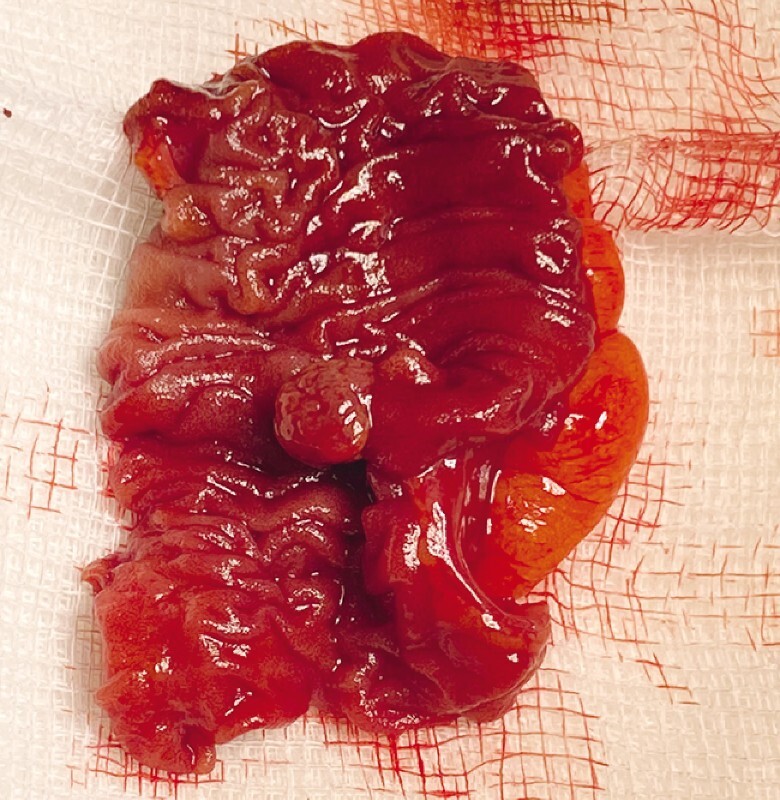
The small bowel surgical resection specimen showing the lesion within the ileum.

Obscure gastrointestinal bleeding (OGIB) is a challenging condition that accounts for nearly 5 % of all gastrointestinal bleeding cases 1 . Small bowel vascular lesions are the most common cause of OGIB 2 . Advances in small bowel capsule endoscopy and device-assisted enteroscopy revolutionized the diagnosis and management of small bowel bleeding 3 . Although the majority of cases can be managed endoscopically, this case highlights the value of intraoperative enteroscopy for the localization and treatment of small bowel lesions when endoscopic treatment is not feasible 4 .

Endoscopy_UCTN_Code_CCL_1AC_2AC

## Notes

Endoscopy E-Videos

https://eref.thieme.de/e-videos
*E-Videos* is an open access online section of the journal *Endoscopy* , reporting on interesting cases and new techniques in gastroenterological endoscopy. All papers include a high-quality video and are published with a Creative Commons CC-BY license. Endoscopy E-Videos qualify for HINARI discounts and waivers and eligibility is automatically checked during the submission process. We grant 100% waivers to articles whose corresponding authors are based in Group A countries and 50% waivers to those who are based in Group B countries as classified by Research4Life (see: https://www.research4life.org/access/eligibility/ ). This section has its own submission website at https://mc.manuscriptcentral.com/e-videos
